# Treatment preferences and their determinants among adults with depression or anxiety in out-patient mental healthcare: systematic review

**DOI:** 10.1192/bjo.2025.10849

**Published:** 2025-10-01

**Authors:** Lara Lenz, Hans-Helmut König, Melanie Leitner, André Hajek

**Affiliations:** Department of Health Economics and Health Services Research, University Medical Centre Hamburg-Eppendorf, Hamburg Centre for Health Economics, Hamburg, Germany

**Keywords:** Preferences, outpatient treatment, psychotherapy, depressive disorders, anxiety or fear-related disorders

## Abstract

**Background:**

Accommodation of treatment preferences is known to improve treatment outcomes and increase patient satisfaction, and is further advised in several national guidelines.

**Aims:**

The aim of this study was to systematically review studies that elicited treatment preferences and related determinants among adults with depressive or anxiety disorder for out-patient mental healthcare.

**Method:**

The systematic review was registered in PROSPERO (CRD42024546311). Studies were retrieved from Web of Science, PubMed, CINAHL and PsycINFO. We included studies of all types that assessed treatment preferences of adults with depressive or anxiety disorder for out-patient care. Extracted data on preferences and determinants were summarised and categorised. Preferences were categorised into treatment approaches, psychotherapy delivery and setting, and psychotherapy parameters. Study quality was assessed with the Mixed-Methods Appraisal Tool.

**Results:**

Nineteen studies were included in the review. Preferences examined related to treatment approaches (*n* = 13), psychotherapy delivery and setting (*n* = 10), and psychotherapy parameters (*n* = 7). High heterogeneity in statistical methods and preference types restricted the derivation of robust conclusions, but tendencies toward a preference for psychotherapy (compared with medication), and particularly individual and face-to-face therapy, were observed. Regarding determinants, results were highly diverse and many findings were derived from single studies.

**Conclusions:**

Our review synthesised evidence on treatment preferences and related determinants in out-patient mental healthcare. Results showed considerable heterogeneity regarding preference types, determinants and statistical methods. We highly recommend to develop and use standardised instruments to assess treatment preferences. Care providers should consider preference variance among patients, and provide individualised care.

In the past decades, there has been a significant increase in the prevalence of depressive disorders^1,2^ and anxiety disorders.^3^ According to the World Health Organization,^4^ depressive and anxiety disorders together make up approximately 60% of all mental disorders worldwide. Depression and anxiety are associated with reduced quality of life,^5,6^ impaired role functioning^7^ and increased mortality risks.^8^ Besides the individual consequences, mental disorders are accompanied by an immense economic burden. According to a systematic review including studies from 48 countries, annual societal costs per patient range from US$1180 to 18 313 (adjusted for inflation and the country’s power parity rate to the USA price level) depending on the mental disorder, whereas depressive and bipolar disorders are associated with annual societal cost per patient of US$5703.^9^ Still, very few patients receive adequate treatment, which is reflected by notable treatment gaps for both depressive and anxiety disorders.^10,11^ Additionally, treatment refusal and premature termination are common in treatment of mental disorders. In their meta-analysis, Swift et al^12^ found an overall treatment refusal rate of 8.2% and an overall premature termination rate of 21.9%.

A main issue that arises when the demand cannot be met by the system is the long waiting times for psychotherapy. For example, in Germany in 2017, a structural reform was made to improve access to out-patient psychotherapy. However, a pre-post evaluation of this reform showed that the time between the initial contact with a psychotherapist and the start of therapy became even longer.^13^ The sum of those findings suggests that the mental healthcare system is used to full capacity. Efficient allocation of resources is an important driver to secure mental healthcare in the future across all countries. In 2001, the Institute of Medicine introduced six aims to improve healthcare quality.^14^ One of those aims is patient-centredness. Per definition, supplying patient-centred healthcare includes acknowledging the patient’s needs, values and preferences, and adapting care accordingly. Patient preferences are defined as the choices an individual makes on different treatment options and characteristics.^15^ In various guidelines across many countries, the integration of patient preferences in the decision-making process of mental healthcare is already highly advised.^16–18^ Research indicates that accommodating treatment preferences increases patient satisfaction^19^ and enhances health outcomes and treatment adherence.^20^

In the light of an expected increase in prevalence, an overwhelmed mental healthcare system and the positive effects of integration of preferences, efficient patient-centred care is gaining in importance, which is reflected by previous research. For example, Tünneßen et al^21^ conducted a systematic review of discrete choice experiments on treatment preferences in patients with depressive or anxiety disorder, including studies that were published before April 2019. They discovered that, in general, process and cost attributes were more important to patients than outcome attributes. To build on those findings, we chose to include all study types in our review. Furthermore, we add value to existing research by examining preferences for various treatment approaches as well as treatment modalities, and review determinants that influence those preferences, which, to our knowledge, has not been done before. Therefore, we seek to address the following research questions: (a) What treatment preferences do adults with symptoms of depressive or anxiety disorder express with regard to out-patient mental healthcare? and (b) What determinants influence these preferences?

Such knowledge is important because research shows that resources in mental healthcare are not optimally allocated. By gaining deeper insights into mental health patients’ preferences, treatment for depressive and anxiety disorders can be further individualised. This contributes to a more patient-centred mental healthcare and improves treatment outcomes and satisfaction.

## Method

We conducted a systematic review on treatment preferences and their determinants among adults with depressive or anxiety disorder in out-patient mental healthcare. The review was registered in PROSPERO (identifier CRD42024546311). Extending PROSPERO, we further explored determinants of preferences, if examined. Structure and content of the review are consistent with the Reporting Guidelines for Meta-analyses of Observational Studies (MOOSE)^22^ and the most recent version of the Preferred Reporting Items for Systematic Reviews and Meta-Analyses (PRISMA) Statement.^23,24^ The completed PRISMA checklist can be found in Supplementary Material D available at https://doi.org/10.1192/bjo.2025.10849. As this was a systematic review of published studies, informed consent and ethical approval were not required.

### Search strategy

First, the search string was created by first author L.L. Second, a librarian from the University Medical Centre Hamburg-Eppendorf was consulted for further inspection of the search string and chosen databases. The librarian agreed on and endorsed our search strategy, so no changes were made. The final search included the following keywords and was applied in Web of Science, PubMed, PsycINFO and CINAHL on 16 May 2024 (see Supplementary Material A for the exact search terms and strings): preferences, patient preferences, depression, depressive disorder, anxiety, anxiety disorder and treatment.

Studies were included if they fulfilled all of the following inclusion criteria: (a) peer-reviewed primary qualitative, quantitative or mixed-methods study; (b) adults (aged 18 years and older); (c) symptoms of depressive or anxiety disorder (clinically diagnosed or self-reported); and (d) out-patient care. For simplicity and readability reasons, in this paper, we will refer to the term ‘disorder’, even if diagnostic criteria may not be fulfilled in all patients. Studies were excluded if they met one or more of the exclusion criteria: (a) review or meta-analysis, (b) in-patient care, (c) secondary depression/anxiety (including perinatal depression/anxiety), (d) veterans and (e) studies comparing different dosing schemes of pharmacological treatment. Furthermore, the search was restricted to articles in English or German. No restrictions were made regarding year of publication or geographical location. We did not review grey literature. Title, abstracts and full texts were screened in a three-step-process in duplicate (by L.L. and M.L.). Both researchers conducted a pre-screening to ensure conformity with inclusion and exclusion criteria. As no clarifications or adjustments had to be made after 20 titles, we further continued to screen independently (deviating from the intended pre-screening of 100 titles as stated in PROSPERO). Any conflicts throughout the screening process were resolved through discussion between L.L. and M.L. No third reviewer (A.H.) had to be consulted.

### Data extraction and synthesis

Starting on 17 June 2024, data extraction was performed by L.L. and carefully checked by M.L. Therefore, a sheet was created *a priori* that contained fields for study characteristics (e.g. sample size, disorder), methods (e.g. preference elicitation method) and results (i.e. preferences and determinants) that were filled for each study. If an article included both anxiety disorder and depression, we treated each disorder as a separate study and extracted data for each disorder. Results from the preference elicitations were described narratively and, where possible, frequencies and coefficients were extracted. If missing, summary statistics were carefully computed or converted manually. Preference types were grouped into three main categories with two subcategories each: (a) treatment approaches (psychotherapy versus pharmacotherapy, other treatment types), (b) psychotherapy delivery and setting (delivery, setting), and (c) psychotherapy parameters (frequency, provider). Each study was assigned at least one of those main categories. Frequencies for each main and subcategory were calculated. Determinants were presented if they were significantly associated with certain preferences in at least one study. The final set of determinants and the signs of the effects or associations are displayed in a table. Any inconsistencies in terms of data extraction and categorisation were planned to be discussed and resolved between L.L., M.L. and A.H., but none occurred.

### Quality assessment

The quality of included studies was assessed with the Mixed Methods Appraisal Tool,^25^ which is commonly used in reviews and enables the appraisal of various study types and designs. For each study design there are five questions regarding the sample, appropriateness of methods and risk of bias that can be answered with *yes*, *no* or *can’t tell*. The assessment was performed independently by two researchers (L.L. and M.L.). Disagreements occurred in 15% of the questions and were resolved through discussion. An overall score is calculated for all questions answered with y*es* and displayed in percentages for each study (20%, 40%, 60%, 80% or 100%). The quality assessment does not result in exclusion of studies.

## Results

**Fig. 1:**
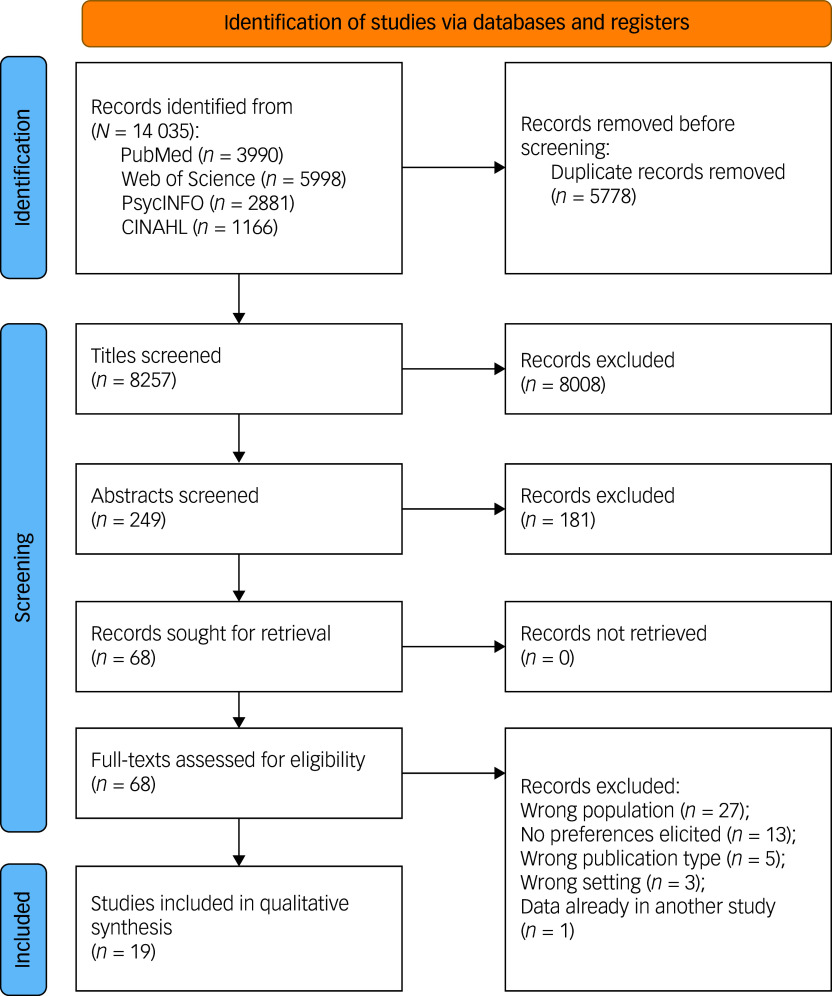
PRISMA flow diagram.

Figure 1 depicts the PRISMA flow diagram^24^ for the study selection process. The electronic search of PubMed, Web of Science, PsycINFO and CINAHL resulted in 14 035 identified studies (see Fig. 1). After removing duplicates, 8257 studies remained. After screening the titles, 8008 records were excluded. We screened the abstracts of the remaining 249 studies and excluded another 181 studies. In total, 68 studies were then screened in full text and 19 studies were included in the qualitative synthesis of the review.^26–44^ The reasons for exclusion of full texts were wrong population (i.e. mental disorder other than depression or anxiety, *n* = 27), no preferences were assessed (*n* = 13), wrong publication type (e.g. conference presentation, *n* = 5), wrong setting (*n* = 3) or same data was already reported in another included study (*n* = 1). In the latter case, the more detailed article was chosen to be included.^30^

### Study characteristics

The study characteristics are displayed in Table 1. The included studies were conducted in Europe (*n* = 8, five in Germany^26,29,30,40,41^ and three in the Netherlands^34,38,39^), North America (*n* = 8, six in the USA^31–33,35,37,42^ and two in Canada^36,44^) and Oceania (*n* = 3, all of them in Australia^27,28,43^). The majority of the studies was published between 2010 and 2019. All of the studies were cross-sectional, 17 studies were quantitative^26–39,41–43^ and two used a mixed-methods approach.^40,44^ Self-administered online or paper-and-pencil questionnaires were more frequently used than interviews. The total sample size equalled 6640 and ranged from 60 to 1602 participants in the individual studies. Most of the studies included about 100–400 participants. In studies including both men and women, proportion of women ranged from 46.6^36^ to 92.3%.^43^ One study included only women^28^ and another study only men.^31^ On average, participants were middle aged in most studies, i.e. between 40 and 50 years old. In studies using online questionnaires, respondents were mainly somewhat younger (e.g. Lokkerbol et al^38^). Two studies were published during or after the COVID-19 pandemic, but did not report the exact period of data collection.^27,43^ In all other studies, data collection was performed before the pandemic.

**Table 1:** Study characteristics

Author (year)	Region	Mode of survey administration	Sample size *n*	Age, years, mean (s.d.)/% of age groups	Proportion female, %	Assessment of disorder	Quality assessment (MMAT) in %
Depression							
Backenstrass et al (2006)^26^	Germany	Paper-and-pencil questionnaire	113^a^	MDD: 42.4 (s.d.: 16.1) SSD: 45.6 (s.d.: 17.8)	MDD: 87.5 SSD: 70.8	PHQ-9 (MDD ≥ 5, SSD ≥ 2)	40
Boehlen et al (2016)^29^ ^b^	Germany	Structured interview and paper-and-pencil questionnaire	240	55–64: 24.6% 65–74: 51.8% 75–84: 23.5%^c^	52.2^c^	PHQ-D (minor ≥ 2, major ≥ 5)	40
Dorow et al (2018)^30^	Germany	Paper-and-pencil questionnaire	641	43.9 (s.d.: 13.8)	68.6	Diagnosis from general practitioner and PHQ-D	80
Dwight-Johnson et al (2000)^33^	USA	Paper-and-pencil questionnaire	1187	44 (s.d.: 15)	71.0	CIDI and CES-D	80
Dwight-Johnson et al (2010)^32^	USA	Interview-led survey	339	49.8 (s.d.: 12.6)	84.0	PHQ-9 and Primary Care Evaluation of Mental Disorders	60
Dwight Johnson et al (2013)^31^	USA	Questionnaire (mode of administration not described)	63^d^	49% were between 60 and 64	0	PHQ-2, SCID for DSM-IV, question on chronic depression, self-report for receipt of diagnosis	80
Groenewoud et al (2015)^34^	The Netherlands	Online questionnaire	368	41 (s.d.: 10.9)	77.0	Questionnaire for DSM-IV-TR and BDI-II	100
Gum et al (2006)^35^	USA	Computer-assisted personal interview	1602^e^	71.1 (s.d.: 7.4)	67.0	SCID for DSM-IV and Hopkins Symptom Checklist-90	80
Houle et al (2013)^36^	Canada	Questionnaire (mode of administration not described)	88	42 (s.d.: 12.2)	46.6	PHQ-9 ≥ 10	20
Khalsa et al (2011)^37^	USA	Paper-and-pencil questionnaire	151^e^	Participants were between 18 and 70	60.3	SCID for DSM-IV and Hamilton Rating Scale for Depression	40
Lokkerbol et al (2019)^38^	The Netherlands	Online questionnaire	165	18–24: 41% 25–30: 16% 31–40: 16% 41–50: 10% 51–60: 13% ≥61: 4%	90	Self-report for current or recent treatment for depression in the past 12 months	40
Löwe et al (2006)^40^	Germany	Telephone interview	87^f^	41.0 (s.d.: 13.6)	66	SCID for DSM-IV and PHQ-9	80
Luck-Sikorski et al (2017)^41^	Germany	Structured face-to-face interview	379^g^	Mild: 81.3 (s.d.: 4.8) Moderate: 81.4 (s.d.: 5.1)	Mild: 71.5 Moderate: 71	Short version GDS (mild ≥ 4, moderate ≥ 6)	80
Raue et al (2009)^42^	USA	Structured interview and questionnaire	60	51.2 (s.d.: 17.4)	78.0	SCID for DSM-IV and Hamilton Rating Scale for Depression	60
Smith et al (2021)^43^	Australia	Online questionnaire	182	36.8 (s.d.: 11.9)	92.3	PHQ-9 ≥ 10	20
Anxiety							
Basile et al (2024)^27^	Australia	Online questionnaire	127	29.17 (s.d.: 11.9)	80.3	Self-report DSM V and GAD-7 ≥ 10	20
Black et al (2023)^28^	Australia	Online questionnaire	99	34.9 (s.d.: 11.3)	100	SIAS-6 ≥ 7 and SPS-6 ≥ 2	20
Boehlen et al (2016)^29^ ^b^	Germany	Structured interview and paper-and-pencil questionnaire	409	55–64: 24.6% 65–74: 51.8% 75–84: 23.5%^c^	52.2^c^	GAD-7 ≥ 5	40
Lokkerbol et al (2019)^39^	The Netherlands	Online questionnaire	126	18–24: 38.9% 25–30: 19.8% 31–40: 13.5% 41–50: 11.1% 51–60: 15.1% ≥61: 1.59%	89.7	Self-report for current or recent treatment for anxiety in the past 12 months	40
Soucy and Hadjistavropoulos (2017)^44^	Canada	Online questionnaire	116	41.3 (s.d.: 14.9)	69.8	Whiteley Index ≥ 5	60

MMAT, Mixed Methods Appraisal Tool; MDD, major depressive disorder; SSD, subsyndromal depression; PHQ, Patient Health Questionnaire; PHQ-D, Patient Health Questionnaire-Depression; CIDI, Composite International Diagnostic Interview; CES-D, Center for Epidemiological Studies Depression Scale; SCID, Structured Clinical Interview for DSM Disorders; BDI-II, Beck Depression Inventory-II; GDS, Geriatric Depression Screening Scale; GAD-7, Generalised Anxiety Disorder Screener; SIAS, Social Interaction Anxiety Scale, SPS, Social Phobia Scale.

a . Proportionate sample of participants with MDD or SSD.

b . Boehlen et al (2016)^29^ is one study (results are reported for both disorders separately).

c . Age and proportion of women only calculated for the whole study sample.

d . Proportionate sample of participants included in conjoint analysis.

e . Proportionate sample of participants who expressed a preference for either psychotherapy or antidepressants.

f . Proportionate sample of participants with depression.

g . Proportionate sample of patients with mild or moderate depressive symptoms.

Depression alone was examined in 14 studies,^26,30–38,40–43^ anxiety disorder alone in four studies^27,28,39,44^ and one study observed both disorders.^29^ Both disorders were mostly assessed with multiple measures (see Table 1). For depression, the most common ones used were different versions of the Patient Health Questionnaire (PHQ),^26,29–32,36,40,43^ followed by the DSM-IV criteria.^31,34,35,37,40,42^ Anxiety was measured with the Generalised Anxiety Disorder Scale-7 (GAD-7),^27,29^ DSM-V criteria,^27^ the Social Interaction Anxiety Scale,^28^ the Social Phobia Scale^28^ or the 14-item Whiteley Index.^44^

#### Quality assessment

The calculated total score of the quality assessment is displayed in Table 1. A more detailed description of the assessment can be found in Supplementary Material B. The overall quality of the included studies was moderate (see Table 1). Only one study fulfilled all five quality criteria and scored 100%,^34^ indicating a high quality, whereas four studies fulfilled only one criterion and scored 20%,^27,28,36,43^ indicating a poor quality. The main concerns among all studies were related to the risk of non-response bias, especially when online surveys were conducted (e.g. Lokkerbol et al^38^). Further issues refer to the elicitation of preferences, as most studies neither used an established instrument nor based their choice of questions and attributes on literature or qualitative evidence. Additionally, some studies used very basic statistical methods to calculate the preferences and reported only means or frequencies (e.g. Basile et al,^27^ Boehlen et al^29^).

### Preference elicitation methods

Preferences were elicited with various methods, such as simple (single or multiple choice) questions with predefined options, open-ended questions, rating tasks or choice tasks (see Table 2 and 3). Most commonly, simple questions with predefined options were used (*n* = 9^26,29,33,35–37,42–44^), either asking for the preferred treatment option (i.e. single choice; e.g. Soucy and Hadjistavropoulos^44^) or asking what treatment options would be taken into consideration (i.e. multiple choice; e.g. Boehlen et al^29^). In one study,^40^ participants were asked open-ended questions to assess their preferred treatment and their responses were grouped into ten preference categories. Five studies applied choice experiments to elicit the respondents’ preferences.^31,32,34,38,39^ These studies included up to ten attributes.^34^ Two studies with choice experiments applied multiple surveys in the same sample, with each consisting of four different attributes.^31,32^ Furthermore, some studies used rating tasks (i.e. Likert scales) to assess the preference strength for different treatment options. One study used solely rating tasks,^30^ whereas three studies combined rating tasks with simple single choice questions.^27,28,41^ Only four studies provided patients with education about the treatments, including a description, benefits and disadvantages, before assessing their preferences.^31,32,36,44^

**Table 2:** Preference attributes for depressive disorders

Author (year)	Preference elicitation method	Treatment approaches		Psychotherapy delivery and setting		Psychotherapy parameters	
		Psychotherapy versus pharmacotherapy	Other treatment types (e.g. physiotherapy, talking with friends/family)	Psychotherapy delivery mode (e.g. in person, internet-based)	Psychotherapy setting (i.e. individual versus group)	Psychotherapy frequency and intensity	Provider of psychotherapy
Backenstrass et al (2006)^26^	Forced choice question	56.6% would consider both, 19.9% only pharmacological treatment, 19.7% only psychotherapy and 13.7% neither	NA	NA	NA	NA	NA
Boehlen et al (2016)^29^	Multiple choice question with 12 options in total^a^	18.3% would consider psychotherapy, 14.6% would consider pharmacological treatment	Three most preferred options: 1. Rehabilitation programme^b^: 43.8% 2. Physiotherapy: 42.1% 3. Alternative medicine: 30.4%	NA	NA	NA	NA
Dorow et al (2018)^30^	5-point Likert scale with eight options in total^c^	58% would consider psychotherapy, 39% would consider medication	Three most preferred options: 1. Psychotherapy: 58% 2. Talking with friends/family: 55% 3. Exercising: 51%	NA	NA	NA	NA
Dwight Johnson et al (2000)^33^	Single choice question with five options^d^	Of those reporting a preference, 32.7% prefer medication and 67.3% prefer psychotherapy	17% prefer no treatment at all	NA	Of those reporting a preference, 35.5% prefer individual counselling and 31.8% prefer group counselling	NA	NA
Dwight Johnson et al (2010)^32^	Conjoint analysis with fractional factorial design (four attributes with two to three levels each)	Counselling was preferred over medication (OR_counselling_ = 1.11, 95% CI: 0.87–1.42; OR_med_ = 0.39, 95% CI: 0.33–0.46; reference: counselling and medication)	NA	NA	No differences for individual versus group treatment (OR_individual_ = 1.07, 95% CI: 0.89–1.28; reference: group)	NA	Primary care preferred over specialty care (OR_primary_ = 1.51, 95% CI: 1.16–2.19; reference: speciality care)
Dwight Johnson et al (2013)^31^	Conjoint analysis with fractional factorial design (seven attributes with two to three levels each)	Medication preferred over counselling (OR_medication_ = 1.61, 95% CI: 1.09–2.37; reference: counselling)	NA	Treatment via telephone preferred over no availability of telephone sessions (OR_available_ = 1.77, 95% CI: 1.18–2.65; reference: not available)	*NA*	No differences regarding frequency (OR_biweekly_ = 0.99, 95% CI: 0.68–1.43; OR_weekly_ = 1.34, 95% CI: 0.92–1.96; reference: monthly)	Psychiatrist preferred over social worker (OR_psychiatrist_ = 2.03, 95% CI: 1.27–3.25; reference: social worker)
Groenewoud et al (2015)^34^	Discrete choice experiment (ten attributes with three levels each)	NA	NA	NA	NA	NA	Medical doctor less preferred than social-psychiatric nurse (OR_doctor_ = −0.22, 95% CI: −0.34 to −0.09, reference: social-psychiatric nurse); having not a good relationship with provider was less preferred than having no relationship (OR_bad_ = −0.64, 95% CI: −0.77 to −0.52), reference: no relationship)
Gum et al (2006)^35^	Forced choice question	57% prefer counselling, 43% prefer antidepressant medication	NA	NA	NA	NA	NA
Houle et al (2013)^36^	Forced choice question (psychotherapy, antidepressants, none)	40.9% prefer psychotherapy, 30.7% prefer antidepressants, 28.4% prefer none	NA	NA	NA	NA	NA
Khalsa et al (2011)^37^	Forced choice dichotomous question	59% prefer psychotherapy, 41% prefer medication	NA	NA	NA	NA	NA
Lokkerbol et al (2019)^38^	Discrete choice experiment (four attributes with three levels each)	NA	NA	Face-to-face preferred over digital treatment and combination of face-to-face + digital preferred over digital only (*β*_digital_ = −1.98, 95% CI: −2.34 to −1.61; *β*_combination_ = 0.46, 95% CI: 0.32 to 0.61; reference: face-to-face)	Individual treatment preferred over small and large groups; small groups preferred over large groups (*β*_small_ = −0.10, 95% CI: −0.22 to 0.03; *β*_large_ = −1.21, 95% CI: −1.49 to −0.94; reference: individual)	Once a week preferred over twice a week (*β*_twice_ = −0.13, 95% CI: −0.27 to 0.08; reference: once a week)	NA
Löwe et al (2006)^40^	Open-ended question via telephone interview	29% would prefer psychotherapy, 6% would prefer medication	Most mentioned treatment options: 1. Psychotherapy: 29% 2. No treatment: 25% 3. Improvement of medical care (e.g. more time to communicate with physician): 22%	NA	NA	NA	NA
Luck-Sikorski et al (2017)^41^	5-point Likert scale with nine options in total^e^ and indication of first and second choice	Medication as first choice preferred over psychotherapy	Medication most common first choice; most common pattern of first and second choice was medication-psychotherapy (10%) followed by medication-talking to family and friends (10%)	NA	NA	NA	NA
Raue et al (2009)^42^	Forced choice with eight options^f^ and forced dichotomous choice (individual psychotherapy versus medication)	70% prefer individual psychotherapy, 30% prefer antidepressant medication	Most common first choice: 1. Individual psychotherapy: 55% 2. Antidepressants: 17% 3. Combined treatment: 17%	NA	Individual psychotherapy (55%) more frequently chosen than group treatment (2%)	NA	NA
Smith et al (2021)^43^	Single choice question with six options referring to CBT^g^	NA	NA	Face-to-face (69.1%) preferred over remote treatment (21.2%): for remote treatment, low intensity (i.e. internet-based or bibliography-based) (94.3%) was preferred over high intensity (i.e. internet-videoconferencing) (5.7%)	NA	For face-to-face treatment, one session per week (85.1%) was preferred over two sessions per week (14.9%)	NA

NA, Not assessed; CBT, cognitive–behavioural therapy.

a . Options: regular consultations with GP, rehabilitation programme, psychotherapy, self-help group, pharmacological treatment, relaxation techniques, more time for consultations with GP, alternative medicine, more information/education, physiotherapy, nothing.

b . Not further specified.

c . Options: medication, psychotherapy, combined treatment, alternative therapies, talk with friends and family, exercise, self-help literature, internet-based self-help programmes.

d . Options: 1. Free medication daily for 6 months, often causes nausea and headaches, 75% chance of cure; 2. Medication daily for 6 months, no or only minor side effects, costs you $80/month ($480 total), 75% chance of cure; 3. Individual counselling 1 h per week for 3 months, costs you $25 a session ($300 total), 75% chance of cure; 4. Group counselling 1 h per week for 3 months, costs you $5 per session ($75 total), 75% chance of cure; 5. Wait and see (no treatment, no cost), 40% chance of cure.

e . Options: medication, psychotherapy, combined treatment, alternative approaches, talking to family and friends, exercise, self-help books, self-help groups, I do not know.

f . Options: antidepressants, individual psychotherapy, group psychotherapy, combined treatment, herbal remedies, religious/spiritual activities, exercise, do nothing.

g . Options: 1. standard weekly face-to-face contact (once a week); 2. accelerated face-to-face treatment (twice a week); 3. internet videoconferencing; 4. low intensity intervention (non-face-to-face); 5. other treatment; 6. none.

**Table 3:** Preference attributes for anxiety disorders

Author (year)	Preference elicitation method	Treatment approaches		Psychotherapy delivery and setting		Psychotherapy parameters	
		Psychotherapy versus pharmacotherapy	Other treatment types (e.g. physiotherapy, talking with friends/family)	Psychotherapy delivery mode (e.g. in person, internet-based)	Psychotherapy setting (i.e. individual versus group)	Psychotherapy frequency and intensity	Provider of psychotherapy
Basile et al (2024)^27^	10-point Likert scale for six delivery and setting options; forced choice for three frequency options	NA	NA	In-person individual treatment (mean 7.59, s.d. 2.86) preferred over remote treatment via video-conferencing (mean 4.31, s.d. 3.55)	In-person individual treatment (mean 7.59, s.d. 2.86) was preferred over in-person group treatment (mean 2.04, s.d. 2.69)	67.9% preferred traditional weekly sessions over long period, 16.5% preferred two to three sessions per week over shorter amount of time, 15.6% preferred brief version (half the time of standard treatment)	NA
Black et al (2023)^28^	Forced choice and 100-point Likert scale for six delivery and setting options	NA	NA	Low-intensity treatment options (i.e. internet-based or bibliography-based; 12.5%) were preferred over high-intensity treatment options (i.e. video-based; 4.2%)	Individual treatment (69.8%) was preferred over group treatment (5.2%)	NA	NA
Boehlen et al (2016)^29^	Multiple choice question with 12 options in total^a^	15.7% would consider psychotherapy, 11.7% would consider pharmacological treatment	Three most preferred options: 1. Physiotherapy: 43.5%; 2. Rehabilitation programme^a^: 40.1%; 3. Alternative medicine: 28.4%	NA	NA	NA	NA
Lokkerbol et al (2019b)^39^	Discrete choice experiment (four attributes with three levels each)	NA	NA	Face-to-face preferred over digital treatment; combination of face-to-face + digital preferred over digital only (*β*_digital_ = −2.12, 95% CI: −2.59 to −1.64; *β*_combination_ = 0.44, 95% CI: 0.25 to 0.63; reference: face-to-face)	Individual treatment preferred over small and large groups; small groups preferred over large groups (*β*_small_ = −0.20, 95% CI: −0.38 to −0.17; *β*_large_ = −1.21, 95% CI: −1.54 to −0.88)	Once a week preferred over twice a week (*β*_twice_ = −0.25, 95% CI: −0.43 to −0.08; reference: once a week)	NA
Soucy and Hadjistavropoulos (2017)^44^	Single choice question with three options^b^	Medication was preferred over ICBT (*p*<0.016)	NA	CBT was preferred over ICBT (*p*<0.016)	NA	NA	NA

NA, Not assessed; CBT, cognitive–behavioural therapy; ICBT, internet-based–cognitive behavioural therapy.

a . Options: regular consultations with GP, rehabilitation programme, psychotherapy, self-help group, pharmacological treatment, relaxation techniques, more time for consultations with GP, alternative medicine, more information/education, physiotherapy, nothing.

b . Options: CBT, ICBT, medication.

### Preference attributes

The preferences extracted from the included studies were categorised into treatment approaches, psychotherapy delivery and setting, and psychotherapy parameters. They are displayed in Table 2 for depressive disorders and Table 3 for anxiety disorders and will be further explained in the following sections. Fourteen studies explored preferences for the choice between psychotherapy and pharmacotherapy (12 in depression studies, two in anxiety studies). Other treatment approaches were investigated in seven studies (six in depression studies and one in anxiety studies). Regarding psychotherapy delivery and setting, preferences for delivery were studied in three depression studies and four anxiety studies, and preferences for the setting were investigated in four depression studies and three anxiety studies. Frequency and intensity preferences were assessed in five studies (three depression and two anxiety studies), and provider preferences were only studied in three depression studies. Thus, in total, in depression studies, treatment approaches were by far the most explored attributes, followed by delivery and setting attributes and psychotherapy parameters. In anxiety studies, delivery and setting attributes were most assessed, followed by treatment approaches and psychotherapy parameters, whereby no anxiety study investigated provider preferences.

#### Treatment approaches

##### Depression

In studies investigating preferences for depression treatment, preferences for psychotherapy versus pharmacological treatment were compared in 12 out of 15 studies. The findings show that psychotherapy was preferred over medication in all but two studies. In studies using a forced choice dichotomous question, psychotherapy was always preferred over medication, with shares between 57 *v*. 43%^35^ and 70 *v*. 30%.^42^ In other studies using single or multiple choice questions or ratings tasks, the share of respondents considering psychotherapy was always larger than the share considering pharmacological treatment. For example in Löwe et al,^40^ 29% would prefer psychotherapy and only 6% would choose medication as their preferred treatment. In contrast to this, two studies reported stronger preferences for medication compared with psychotherapy.^26,31^ In Backenstrass et al^26^ more than half of the respondents (56.6%) stated they would consider both treatment options, 19.9% would consider only pharmacological treatment and 19.7% would only consider psychotherapy. In another study applying conjoint analysis, medication was also preferred over counselling (odds ratio 1.61, 95% CI 1.09–2.37).^31^

Regarding other treatment approaches, in six studies, participants considered further treatment options apart from psychotherapy or pharmacotherapy. For example, talking to friends and family was among the most common top three choices in two studies.^30,41^ In Boehlen et al,^29^ the most preferred treatment option was rehabilitation programme (43.8%), followed by physiotherapy (42.1%) and alternative medicine (30.4%). However, the option rehabilitation programme was not further specified to respondents. Psychotherapy and pharmacological treatment were the seventh and ninth choice among the most preferred treatment methods. Moreover, in one study, a preference for a combined treatment was as common as a preference for antidepressant medication.^42^ In three studies, about one-fourth to one-fifth of respondents chose no treatment over any kind of active treatment,^29,33,40^ whereas in two other studies, treatment was refused by no one^42^ or very few respondents (2.4% in Smith et al^43^).

##### Anxiety

In studies eliciting preferences for treatment of anxiety disorder, comparison of preferences for psychotherapy versus pharmacological treatment was performed in two studies. Boehlen et al^29^ reported that 15.7% of respondents with generalised anxiety disorder would consider psychotherapy, whereas 11.7% would consider pharmacological treatment. In another study, preference strength for medication was significantly higher than for internet-delivered cognitive–behavioural therapy, but not in comparison to regular cognitive–behavioural therapy.^44^

Regarding other treatment approaches, only in one study, participants expressed preferences for other treatment approaches besides psychotherapy and pharmacotherapy. In Boehlen et al,^29^ the three most preferred options were physiotherapy (43.5%), rehabilitation programme (40.1%, not further specified) and alternative medicine (28.4%). The share of respondents choosing psychotherapy or medication in this study was 15.7 and 11.7%, respectively.^29^

#### Psychotherapy delivery and setting

##### Depression

In three studies, preferences for delivery modes were assessed. Face-to-face treatment was preferred over digital treatment in two studies,^38,43^ and a combination of face-to-face and digital therapy was preferred over fully digital treatment.^38^ Moreover, Lokkerbol et al^38^ found that the attribute face-to-face versus digital had the highest conditional relative importance in the discrete choice experiment (45.7%). In Dwight Johnson et al,^31^ the option to receive treatment via telephone was preferred over not having this option (odds ratio 1.77, 95% CI 1.18–2.65).

Regarding the setting, individual therapy was preferred over group therapy.^33,38,42^ Additionally, small groups of three to five persons were preferred over large groups of six to ten persons, and this attribute was the second most conditional relative important of all (32.9%).^38^ In one study, individual psychotherapy was the most common first choice (55%) and group therapy was only chosen by 2%,^42^ but this difference was smaller in Dwight-Johnson et al^33^ (35.5 *v*. 31.8%), and another study did not find a significant preference for either individual or group treatment.^32^

##### Anxiety

In accordance with the delivery mode preferences for depression treatment, in studies investigating preferences for anxiety disorder, face-to-face treatment was preferred over digital treatment,^27,39^ and combined face-to-face with digital treatment was preferred over fully digital treatment.^39^ Again, the attribute concerning face-to-face versus digital treatment had the highest conditional relative importance in the discrete choice experiment (47.2%).^39^ Furthermore, low-intensity treatment options (i.e. internet-based or bibliography-based psychotherapy) were preferred over high-intensity treatment options (i.e. video-based psychotherapy).^28^

Regarding setting, individual psychotherapy was preferred over group psychotherapy in all studies assessing this attribute.^27,28,39^ Again, small groups (three to five persons) were preferred over large groups (six to ten persons) (small groups: odds ratio −0.20, 95% CI −0.38 to −0.17; large groups: odds ratio −1.21, 95% CI −1.54 to −0.88; reference: individual) and this attribute had the second highest conditional relative importance (31.8%).^39^

#### Psychotherapy parameters

##### Depression

Concerning frequency and intensity, two studies reported a preference for one session per week over two sessions per week;^38,43^ that is, in Smith et al,^43^ 85% of participants chose standard face-to-face treatment (once a week) and 15% chose the accelerated version (twice a week). In Lokkerbol et al,^38^ two times per week was less preferred than once a week (two times a week: odds ratio −0.13, 95% CI −0.27 to 0.08), and the intensity of treatment had the lowest conditional relative importance among all assessed attributes (5.0%). One study did not find any differences regarding frequency preferences.^31^

Concerning the treatment provider, the findings all derive from studies applying choice experiments. In Dwight Johnson et al,^31^ a psychiatrist was preferred over a social worker as treatment provider (odds ratio 2.03, 95% CI 1.27–3.25), whereas in another study, a medical doctor was less preferred than a social-psychiatric nurse (odds ratio −0.22, 95% CI −0.34 to −0.09).^34^ Furthermore, in one study, primary care was preferred over speciality care.^32^ Regarding the relationship with the treatment provider, Groenewoud et al^34^ found that having no relationship with the provider was preferred over having a not very good relationship with the provider (Poor relationship: odds ratio −0.64, 95% CI −0.77 to −0.52; reference: no relationship).

##### Anxiety

Concerning frequency and intensity, two studies reported preferences for the frequency or intensity of treatment. In one study, treatment once a week was preferred over treatment twice a week (two times a week: odds ratio −0.25, 95% CI −0.43 to −0.08; reference: once a week).^39^ Moreover, Basile et al^27^ assessed three different frequency options: 67.9% preferred traditional weekly sessions over a long period, 16.5% preferred two to three sessions per week over a shorter time period and 15.6% preferred a brief version of treatment (i.e. half the time of standard treatment).

#### Other preferences

Further findings regarding preferences for depression or anxiety treatment were low cost^31,34^ and short waiting times until the onset of treatment.^34,38,39^ The conditional relative importance of waiting time was 16.1% in a study on preferences for anxiety treatment,^39^ and 16.4% in a study on preferences for depression treatment.^38^

### Determinants

Socioeconomic and health-related determinants of treatment preferences will be explained in the following section. If a determinant was significantly associated with treatment preferences in at least one study, it is displayed in a column in Supplementary Table S1. An overview of all investigated determinants per study is provided in Supplementary Material C. Sociodemographic factors were more frequently examined in the included studies than health-related factors.

#### Socioeconomic factors

##### Depression

Regarding age, older respondents were less likely to choose internet-based treatment compared with younger respondents.^30^ Furthermore, one study showed that, in general, older respondents showed more difficulties in choosing their preferred treatment.^41^ Besides age, gender was also found to be associated with preferences. Women preferred psychotherapy over medication^33,35,36^ and individual counselling over group counselling.^33^ Male respondents were less likely to choose alternative treatment (odds ratio 0.63, 95% CI 0.46–0.87) or self-help literature (odds ratio 0.66, 95% CI 0.48–0.90) compared with female respondents.^30^ Being single was also associated with choosing psychotherapy compared with being married.^30^ In the matter of ethnicity, Dwight Johnson et al^31^ found that White men were more likely to choose medication compared with Mexican men, whereas in another study, being Black American was associated with preferring counselling over medication.^33^ Three studies did not find any significant associations between ethnicity and preferences.^35–37^ Groenewoud et al^34^ found that respondents with a higher level of education based their choice on a larger number of attributes compared with respondents with a lower level of education. Moreover, a high education level was associated with a preference for psychotherapy,^30,36^ choosing individual over group therapy^33^ and having an aversion against long waiting times and fully digital treatment.^38^ In one study, wealthy respondents were more likely to choose any kind of active treatment over no treatment (odds ratio 3.74, 95% CI 1.77–7.91).^33^

##### Anxiety

In one anxiety study, younger respondents had less aversion against digital treatment compared with older adults,^39^ whereas another study did not find any significant differences in treatment preferences regarding age.^28^ Additionally, in terms of education, respondents with a higher level of education had a stronger preference for shorter waiting times and a stronger aversion against a treatment intensity of two times per week compared with respondents with a lower level of education.^39^

#### Health-related factors

##### Depression

Although four studies found no significant association between the severity of symptoms and preferences,^26,30,34,38^ other studies reported that a higher depression severity was associated with higher preference for medication (major depression: odds ratio 1.45, 95% CI 1.12–1.86; reference: dysthymia)^35^ and lower endorsement of psychotherapy,^41^ or it was simply reported that symptom severity affected treatment choice.^32^ In terms of comorbidity, one study reported that having a comorbid anxiety disorder was associated with a general preference for active treatment compared with no treatment,^33^ whereas another study reported an association of comorbid anxiety disorder with a preference for alternative treatment.^30^ Regarding the treatment history, one study found that having a treatment history of depression was associated with a lower preference for medication and combined treatment,^30^ and another study reported that having no recent antidepressant treatment was associated with preferring counselling over medication.^33^ Additionally, Gum et al^35^ found that patients who had previously received antidepressant medication or had found it helpful in the past were more likely to prefer medication over psychotherapy, whereas patients who had previously received psychotherapy or had found it helpful in the past were less likely to prefer medication over psychotherapy. In contrast to this, one study reported that participants who preferred psychotherapy had fewer previous courses of psychotherapy compared with respondents preferring medication.^37^ One study explored the association of having a family history of depression and found that respondents with a family history of depression had a stronger preference for psychotherapy compared with respondents without a family history of depression (odds ratio 7.8, 95% CI 1.6–37.7).^36^ Furthermore, having a greater knowledge about medication was associated with preferring active treatment over no treatment, whereas having a greater knowledge about counselling was associated with preferring counselling over medication and individual over group counselling.^33^

##### Anxiety

One study explored the association of having a treatment history of anxiety disorder with treatment preferences and found that respondents who had previously received psychological treatment were more likely to choose individual face-to-face treatment compared with those who had not.^28^

#### Other factors

Three studies found other factors associated with preferences apart from the described sociodemographic and health-related factors (not displayed). For example, in a USA study,^33^ having paid sick leave was associated with preferring counselling over medication (odds ratio 1.59, 95% CI 1.10–2.30). Moreover, in another study, higher empowerment was associated with lower preference for medication and combined treatment, and stronger preference for talking with family and friends and exercising.^30^ Houle et al^36^ found that currently receiving psychotherapy was associated with a strong preference for psychotherapy compared with antidepressant medication (odds ratio 17.3, 95% CI: 2.7–109.3^36^).

## Discussion

Our systematic review synthesised the existing evidence on treatment preferences and their determinants among adults with depressive or anxiety disorder in out-patient mental healthcare. To our knowledge, this is the first systematic review on that topic that includes all study types and designs. However, no qualitative study meeting the eligibility criteria could be identified. Of 19 studies included in this review, four studies examined preferences for anxiety disorders, two focused on both depression and anxiety, and the remaining studies investigated preferences for depressive disorders only. We observed high heterogeneity in terms of the study designs and methods, impeding the formulation of robust conclusions. However, some patterns emerged from the data, suggesting a possible tendency toward preferences for psychotherapy over medication, face-to-face over digital treatment and individual over group therapy.

### Preferences and determinants

Preferences as well as their determinants were heterogeneous, and most findings of this review resulted from single studies. In the following section, we will discuss the three main findings in more detail and renounce the in-depth interpretation of further results deriving from single studies.

#### Preference for psychotherapy (and determinants)

Data synthesis of the study results showed that psychotherapy was preferred over medication in the majority of samples with depressive disorder (nine out of 19 studies). Yet, three studies reported a preference for antidepressant medication. In previous systematic reviews, one with meta-analysis, it has been shown that psychotherapy was preferred over medication,^45,46^ with the main reasons for not choosing pharmacological treatment being fear of side-effects and fear of losing control.^46^ Another meta-analysis^12^ evaluated treatment refusal and premature treatment termination in patients with mental disorders and found that patients with depression were more likely to refuse or drop out of treatment if they received pharmacotherapy compared with psychotherapy. Regarding determinants, our results showed that women in particular^45^ were more likely to choose psychotherapy. It is well known that men are less likely to seek psychotherapy for mental health problems compared with women,^47^ which is often associated with their image of masculinity and feelings of shame.^48^ Moreover, our findings suggest that patients with a higher level of education also tend to prefer psychotherapy.

#### Preference for face-to-face treatment (and determinants)

Digital mental health interventions receive growing attention, especially since the COVID-19 pandemic. Besides equivalent effectiveness of remote psychotherapy and face-to-face treatment,^49^ digital mental health interventions produce lower costs and enable easier access independent from time and place compared with traditional face-to-face-treatment.^50^ However, our results suggested that face-to-face treatment may be preferred over digital treatment (five out of 19 studies). This could be related to numerous factors ranging from person-related barriers (such as lack of familiarity or limited digital literacy) to technology-related barriers (such as restricted access or technical issues).^51^ Data synthesis of determinants indicated that younger respondents were generally more likely to accept and choose digital mental health treatment compared with older respondents. Hence, benefits of digital mental health treatment need to be further promoted to increase acceptance of digital mental health interventions, especially among older adults.

#### Preference for individual therapy (and determinants)

Regarding the setting of psychotherapy, six studies reported a preference for individual therapy compared with group therapy, and there was one study reporting no significant difference for either one of the setting options. A previous meta-analysis revealed that individual treatment was slightly more effective than group treatment in depression,^52^ whereas another meta-analysis showed no significant differences regarding effectiveness in anxiety disorders.^53^ Thus, there must be reasons for the observed preference apart from effectiveness. Benefits (both objective and perceived) of individual therapy compared with group therapy might include higher anonymity and stronger focus on individual needs and values.

### Study quality and future research

Overall, the included studies were of moderate quality, which was mainly because of convenience samples and risk of non-response bias, as well as the applied methods for assessing preferences in the sample.

The majority of studies used online convenience samples for their research. Despite the numerous advantages (e.g. inexpensive, easy and efficient access to the sample) of this sampling strategy, it holds a few severe limitations. Patients who engage actively in online questionnaires might differ from patients who do not, and in most studies, analyses on the difference between respondents and non-respondents usually cannot be made. Hence, selection bias as well as non-response bias cannot be ruled out.

Furthermore, we observed high heterogeneity in the studies regarding their applied methods and investigated preference types. The applied methods ranged from simple calculation of frequencies from single choice questions^37^ to complex choice modelling methods.^38^ Moreover, many studies compared different treatment approaches,^29^ whereas fewer investigated delivery or setting preferences.^43^ The resulting diversity of study results and evidence strength aggravates the data synthesis in systematic reviews. To promote comparability of studies in the future, researchers should use validated instruments (such as the Cooper Norcross Inventory^54^) or develop new tools that include parameters that have not yet been considered in existing instruments.

In only a few studies did patients receive information about the different treatments they could choose from. Future research could study the impact of patient education on treatment preferences and decision-making.

Investigated determinants were also highly diverse and most studies only examined the influence of single determinants. Interactions of multiple determinants or application of latent class analysis would be interesting to detect patterns that contribute to the understanding of preferences and choices in out-patient mental healthcare.

Finally, we only included quantitative and mixed-methods studies, as no qualitative study met the predefined inclusion criteria during the selection process. However, qualitative evidence might contribute to more in-depth results. More precisely, qualitative studies could contribute to possible explanations for the patients’ preferences and choices. Moreover, with findings from qualitative research unobserved treatment attributes that are important for patients’ choice of treatment and provider in out-patient mental healthcare could be identified.

### Strengths and limitations

We want to acknowledge some strengths and shortcomings of this review. To increase transparency and quality, our review was registered in PROSPERO and follows the MOOSE and PRISMA guidelines. Our search term was approved by a librarian from the University Medical Centre Hamburg-Eppendorf and then applied in four databases. No further hand search was conducted. We included only peer-reviewed articled that were published in either English or German. As a result, we may have failed to include relevant articles; however, this choice contributed to the quality of included studies. Also, key steps in study selection and data extraction were performed in duplicate, as well as the quality assessment.

In conclusion, our systematic review summarised studies on treatment preferences and determinants in out-patient mental healthcare. The majority of studies focused on depressive disorders, and only a few investigated preferences of patients with anxiety disorders. The results indicate a tendency to favour psychotherapy over medication for depression treatment. Furthermore, tendencies toward a preference for face-to-face treatment and individual therapy were observed among patients of both depressive and anxiety disorders. However, the determinants of these preferences were primarily derived from single studies or were somewhat inconsistent. It is important to note that the included studies demonstrated considerable heterogeneity in terms of tools, statistical methods and examined preference types, restricting the generation of robust conclusions. This underscores the need for standardised instruments in future research, to enhance comparability and strengthen the evidence for treatment preferences among adults with depressive or anxiety disorder in out-patient mental healthcare. In terms of clinical implications, providers should be aware that preferences can be diverse and cannot be generalised, which highlights the importance of assessment and integration of treatment preferences into individual care planning.

## Supporting information

Lenz et al. supplementary material 1 Lenz et al. supplementary material

Lenz et al. supplementary material 2 Lenz et al. supplementary material

## Data Availability

Data availability is not applicable to this article as no new data were created or analysed in this study.

## Supplementary material

The supplementary material is available online at https://doi.org/10.1192/bjo.2025.10849

## References

[1] Steffen A , Thom J , Jacobi F , Holstiege J , Bätzing J. Trends in prevalence of depression in Germany between 2009 and 2017 based on nationwide ambulatory claims data. J Affect Disorders 2020; 271: 239–47. 32479322 10.1016/j.jad.2020.03.082

[2] Goodwin RD , Dierker LC , Wu M , Galea S , Hoven CW , Weinberger AH. Trends in U.S. depression prevalence from 2015 to 2020: the widening treatment gap. Am J Prev Med 2022; 63: 726–33. 36272761 10.1016/j.amepre.2022.05.014 PMC9483000

[3] Yang X , Fang Y , Chen H , Zhang T , Yin X , Man J , et al. Global, regional and national burden of anxiety disorders from 1990 to 2019: results from the Global Burden of Disease Study 2019. Epidemiol Psychiatr Sci 2021; 30: e36. 33955350 10.1017/S2045796021000275 PMC8157816

[4] World Health Organization (WHO). *World Mental Health Report: Transforming Mental Health for All*. WHO, 2022 (https://www.who.int/publications/i/item/9789240049338).

[5] Hansson L. Quality of life in depression and anxiety. Int Rev Psychiatry 2002; 14: 185–9.

[6] Olatunji BO , Cisler JM , Tolin DF. Quality of life in the anxiety disorders: a meta-analytic review. Clin Psychol Rev 2007; 27: 572–81. 17343963 10.1016/j.cpr.2007.01.015

[7] Druss BG , Hwang I , Petukhova M , Sampson NA , Wang PS , Kessler RC. Impairment in role functioning in mental and chronic medical disorders in the United States: results from the National Comorbidity Survey Replication. Mol Psychiatry 2009; 14: 728–37. 18283278 10.1038/mp.2008.13 PMC2700857

[8] Chesney E , Goodwin GM , Fazel S. Risks of all-cause and suicide mortality in mental disorders: a meta-review. World Psychiatry 2014; 13: 153–60. 24890068 10.1002/wps.20128 PMC4102288

[9] Christensen MK , Lim CCW , Saha S , Plana-Ripoll O , Cannon D , Presley F , et al. The cost of mental disorders: a systematic review. Epidemiol Psychiatr Sci 2020; 29: e161. 32807256 10.1017/S204579602000075X PMC7443800

[10] Alonso J , Liu Z , Evans-Lacko S , Sadikova E , Sampson N , Chatterji S , et al. Treatment gap for anxiety disorders is global: results of the World Mental Health Surveys in 21 countries. Depress Anxiety 2018; 35: 195–208. 29356216 10.1002/da.22711 PMC6008788

[11] Moitra M , Santomauro D , Collins PY , Vos T , Whiteford H , Saxena S , et al. The global gap in treatment coverage for major depressive disorder in 84 countries from 2000-2019: a systematic review and Bayesian meta-regression analysis. PLoS Med 2022; 19: e1003901. 35167593 10.1371/journal.pmed.1003901 PMC8846511

[12] Swift JK , Greenberg RP , Tompkins KA , Parkin SR. Treatment refusal and premature termination in psychotherapy, pharmacotherapy, and their combination: a meta-analysis of head-to-head comparisons. Psychotherapy 2017; 54: 47–57. 28263651 10.1037/pst0000104

[13] Kruse J , Kampling H , Bouami SF , Grobe TG , Hartmann M , Jedamzik J , et al. Outpatient psychotherapy in Germany—an evaluation of the structural reform. Dtsch Arztebl Int 2024; 121: 315–22. 38544323 10.3238/arztebl.m2024.0039 PMC11413774

[14] Institute of Medicine (US) Committee on Quality of Health Care in America. Crossing the Quality Chasm: A New Health System for the 21st Century. National Academies Press (US), 2001 (https://pubmed.ncbi.nlm.nih.gov/25057539/). 25057539

[15] Swift JK , Callahan JL , Vollmer BM. Preferences. J Clin Psychol 2011; 67: 155–65. 21120917 10.1002/jclp.20759

[16] National Institute for Health and Care Excellence. Depression in Adults: Treatment and Management. NICE, 2022 (https://pubmed.ncbi.nlm.nih.gov/35977056/). 35977056

[17] Bandelow B , Aden I , Alpers G , Benecke A , Beutel M , Deckert J , et al. Deutsche S3-Leitlinie Behandlung von Angststörungen, Version 2 [*German S3 Guideline for the Treatment of Anxiety Disorders, Version 2*]. Association of the Scientific Medical Societies in Germany, 2021 (https://register.awmf.org/assets/guidelines/051-028l_S3_Behandlung-von-Angststoerungen_2021-06.pdf).

[18] American Psychological Association . *APA* Clinical Practice Guideline for the Treatment of Depression across Three Age Cohorts . APA, 2023 (https://www.apa.org/depression-guideline/guideline.pdf).

[19] Umar N , Schaarschmidt M , Schmieder A , Peitsch WK , Schollgen I , Terris DD. Matching physicians treatment recommendations to patients’ treatment preferences is associated with improvement in treatment satisfaction. J Eur Acad Dermatol Venereol 2013; 27: 763–70. 22631875 10.1111/j.1468-3083.2012.04569.x

[20] Swift JK , Callahan JL , Cooper M , Parkin SR. The impact of accommodating client preference in psychotherapy: a meta-analysis. J Clin Psychol 2018; 74: 1924–37. 30091140 10.1002/jclp.22680

[21] Tünneßen M , Hiligsmann M , Stock S , Vennedey V. Patients preferences for the treatment of anxiety and depressive disorders: a systematic review of discrete choice experiments. J Med Econ 2020; 23: 546–56. 32011209 10.1080/13696998.2020.1725022

[22] Brooke BS , Schwartz TA , Pawlik TM. MOOSE reporting guidelines for meta-analyses of observational studies. JAMA Surg 2021; 156: 787–8. 33825847 10.1001/jamasurg.2021.0522

[23] Moher D , Liberati A , Tetzlaff J , Altman DG , The PRISMA Group. Preferred reporting items for systematic reviews and meta-analyses: the PRISMA statement. PLoS Med 2009; 6: e1000097. 19621072 10.1371/journal.pmed.1000097 PMC2707599

[24] Page MJ , McKenzie JE , Bossuyt PM , Boutron I , Hoffmann TC , Mulrow CD , et al. The PRISMA 2020 statement: an updated guideline for reporting systematic reviews. PLoS Med 2021; 18: e1003583. 33780438 10.1371/journal.pmed.1003583 PMC8007028

[25] Hong QN , Fàbregues S , Bartlett G , Boardman F , Cargo M , Dagenais P , et al. The Mixed Methods Appraisal Tool (MMAT) version 2018 for information professionals and researchers. Educ Inform 2018; 34: 285–91.

[26] Backenstrass M , Joest K , Frank A , Hingmann S , Mundt C , Kronmuller KT. Preferences for treatment in primary care: a comparison of nondepressive, subsyndromal and major depressive patients. Gen Hosp Psychiatry 2006; 28: 178–80. 16516070 10.1016/j.genhosppsych.2005.10.001

[27] Basile VT , Newton-John T , Wootton BM. Treatment histories, barriers, and preferences for individuals with symptoms of generalized anxiety disorder. J Clin Psychol 2024; 80: 1286–305. 38384113 10.1002/jclp.23665

[28] Black JA , Paparo J , Wootton BM. A preliminary examination of treatment barriers, preferences, and histories of women with symptoms of social anxiety disorder. Behav Change 2023; 40: 267–77.

[29] Boehlen FH , Herzog W , Maatouk I , Saum KU , Brenner H , Wild B. Treatment preferences of elderly patients with mental disorders. Z Gerontol Geriatr 2016; 49: 120–5. 26033574 10.1007/s00391-015-0908-x

[30] Dorow M , Löbner M , Pabst A , Stein J , Riedel-Heller SG. Preferences for depression treatment including internet-based interventions: results from a large sample of primary care patients. Front Psychiatry 2018; 9: 181. 29867605 10.3389/fpsyt.2018.00181 PMC5966543

[31] Dwight Johnson M , Apesoa-Varano C , Hay J , Unutzer J , Hinton L. Depression treatment preferences of older white and Mexican origin men. Gen Hosp Psychiatry 2013; 35: 59–65. 23141027 10.1016/j.genhosppsych.2012.08.003 PMC4041603

[32] Dwight-Johnson M , Lagomasino IT , Hay J , Zhang L , Tang L , Green JM , et al. Effectiveness of collaborative care in addressing depression treatment preferences among low-income Latinos. Psychiatr Serv 2010; 61: 1112–8. 21041350 10.1176/ps.2010.61.11.1112

[33] Dwight-Johnson M , Sherbourne CD , Liao D , Wells KB. Treatment preferences among depressed primary care patients. J Gen Intern Med 2000; 15: 527–34. 10940143 10.1046/j.1525-1497.2000.08035.x PMC1495573

[34] Groenewoud S , Van Exel NJA , Bobinac A , Berg M , Huijsman R , Stolk EA. What influences patients decisions when choosing a health care provider? Measuring preferences of patients with knee arthrosis, chronic depression, or Alzheimer’s disease, using discrete choice experiments. Health Serv Res 2015; 50: 1941–72. 26768957 10.1111/1475-6773.12306 PMC4693843

[35] Gum AM , Areán PA , Hunkeler E , Tang LQ , Katon W , Hitchcock P , et al. Depression treatment preferences in older primary care patients. Gerontologist 2006; 46: 14–22. 16452280 10.1093/geront/46.1.14

[36] Houle J , Villaggi B , Beaulieu MD , Lespérance F , Rondeau G , Lambert J. Treatment preferences in patients with first episode depression. J Affect Disorders 2013; 147: 94–100. 23167975 10.1016/j.jad.2012.10.016

[37] Khalsa SR , McCarthy KS , Sharpless BA , Barrett MS , Barber JP. Beliefs about the causes of depression and treatment preferences. J Clin Psychol 2011; 67: 539–49 21365652 10.1002/jclp.20785

[38] Lokkerbol J , Geomini A , van Voorthuijsen J , van Straten A , Tiemens B , Smit F , et al. A discrete-choice experiment to assess treatment modality preferences of patients with depression. J Med Econ 2019; 22: 178–86. 30501437 10.1080/13696998.2018.1555404

[39] Lokkerbol J , van Voorthuijsen JM , Geomini A , Tiemens B , van Straten A , Smit F , et al. A discrete-choice experiment to assess treatment modality preferences of patients with anxiety disorder. J Med Econ 2019; 22: 169–77. 30501135 10.1080/13696998.2018.1555403

[40] Löwe B , Schulz U , Grafe K , Wilke S. Medical patients attitudes toward emotional problems and their treatment. What do they really want? J Gen Intern Med 2006; 21: 39–45. 16423121 10.1111/j.1525-1497.2005.0266.x PMC1484618

[41] Luck-Sikorski C , Stein J , Heilmann K , Maier W , Kaduszkiewicz H , Scherer M , et al. Treatment preferences for depression in the elderly. Int Psychogeriatr 2017; 29: 389–98. 27890036 10.1017/S1041610216001885

[42] Raue PJ , Schulberg HC , Heo M , Klimstra S , Bruce ML. Patients depression treatment preferences and initiation, adherence, and outcome: a randomized primary care study. Psychiatr Serv 2009; 60: 337–43. 19252046 10.1176/appi.ps.60.3.337 PMC2710876

[43] Smith S , Paparo J , Wootton BM. Understanding psychological treatment barriers, preferences and histories of individuals with clinically significant depressive symptoms in Australia: a preliminary study. Clin Psychol 2021; 25: 223–33.

[44] Soucy JN , Hadjistavropoulos HD. Treatment acceptability and preferences for managing severe health anxiety: perceptions of internet-delivered cognitive behaviour therapy among primary care patients. J Behav Ther Exp Psychiatry 2017; 57: 14–24. 28242411 10.1016/j.jbtep.2017.02.002

[45] McHugh RK , Whitton SW , Peckham AD , Welge JA , Otto MW. Patient preference for psychological vs pharmacologic treatment of psychiatric disorders: a meta-analytic review. J Clin Psychiatry 2013; 74: 595–602. 23842011 10.4088/JCP.12r07757 PMC4156137

[46] van Schaik DJ , Klijn AF , van Hout HP , van Marwijk HW , Beekman AT , de Haan M , et al. Patients preferences in the treatment of depressive disorder in primary care. Gen Hosp Psychiatry 2004; 26: 184–9. 15121346 10.1016/j.genhosppsych.2003.12.001

[47] Rommel A , Bretschneider J , Kroll L. Inanspruchnahme psychiatrischer und psychotherapeutischer Leistungen. Individuelle Determinanten und regionale Unterschiede [Use of psychiatric and psychotherapeutic services. Individual determinants and regional differences]. J Health Monitor 2017; 2: 3–23.

[48] Shepherd G , Astbury E , Cooper A , Dobrzynska W , Goddard E , Murphy H , et al. The challenges preventing men from seeking counselling or psychotherapy. Ment Health Prev 2023; 31: 200287.

[49] Carlbring P , Andersson G , Cuijpers P , Riper H , Hedman-Lagerlof E. Internet-based vs. face-to-face cognitive behavior therapy for psychiatric and somatic disorders: an updated systematic review and meta-analysis. Cogn Behav Ther 2018; 47: 1–18. 29215315 10.1080/16506073.2017.1401115

[50] Woon LS , Maguire PA , Reay RE , Looi JCL. Telepsychiatry in Australia: a scoping review. Inquiry 2024; 61: 469580241237116. 38462906 10.1177/00469580241237116 PMC10929062

[51] Borghouts J , Eikey E , Mark G , De Leon C , Schueller SM , Schneider M , et al. Barriers to and facilitators of user engagement with digital mental health interventions: systematic review. J Med Internet Res 2021; 23: e24387. 33759801 10.2196/24387 PMC8074985

[52] Cuijpers P , van Straten A , Warmerdam L. Are individual and group treatments equally effective in the treatment of depression in adults? A meta-analysis. Eur J Psychiatry 2008; 22: 38–51.

[53] Barkowski S , Schwartze D , Strauss B , Burlingame GM , Rosendahl J. Efficacy of group psychotherapy for anxiety disorders: a systematic review and meta-analysis. Psychother Res 2020; 30: 965–82. 32093586 10.1080/10503307.2020.1729440

[54] Cooper M , Norcross JC. A brief, multidimensional measure of clients therapy preferences: The Cooper-Norcross Inventory of Preferences (C-NIP). Int J Clin Health Psychol 2016; 16: 87–98. 30487853 10.1016/j.ijchp.2015.08.003 PMC6225020

