# Supplementary material for: Treatment preferences and their determinants among adults with depression or anxiety in out-patient mental healthcare: systematic review
Source: BJPsych Open. 2025 Oct 1;11(6):e229. doi: 10.1192/bjo.2025.10849 (PMC12529313; doi:10.1192/bjo.2025.10849)
Supplement: Lenz et al. supplementary material 1 — Lenz et al. supplementary material [file S2056472425108491sup001.docx]

| Author (Year) | Analysis method | | Sociodemographic factors | | | | | |
| --- | --- | --- | --- | --- | --- | --- | --- | --- |
|  |  | | **Age** | **Sex** | **Ethnicity** | **Education** | **Income/Wealth** | **Marital status** |
| *Depression* | | | | | | | | |
| Dorow et al. (2018) | | **Eight ordinal logistic regression models for all treatment options** | Higher age was associated with lower preference for internet-based self-help programs (OR=0.99, 95% CI: 0.98 to 0.99) | Being male was associated with lower preference for alternative treatment (OR=0.63, 95% CI: 0.46 to 0.87) and self-help literature (OR=0.66, 95% CI: 0.48 to 0.90) | *NA* | High education (compared to low education) was associated with preference for psychotherapy (OR=2.27, 95% CI: 1.32 to 3.88), exercise (OR=4.77, 95% CI: 2.73 to 8.35) and internet-based self-help programs (OR=3.10, 95% CI: 1.79 to 5.34) and middle education (compared to low education) was associated with preference for self-help literature (OR=2.06, 95% CI: 1.30 to 3.25) | *NA* | Being single was associated with preference for psychotherapy compared to married respondents (OR=1.82, 95% CI: 1.28 to 2.58) |
| Dwight Johnson et al. (2000) | | **Three nested logistic regression models (active vs no, med vs counselling, individual vs group)** | No significant differences (e.g. proportion choosing counselling over medication, age 17-34: 65.9%, age 35-59: 67.2%, age ≥60: 72.2%) | Being female was associated with preferring counselling over medication (OR=1.45, 95% CI: 1.01 to 2.09) and with choosing individual counselling over group counselling (OR=2.0, 95% CI: 1.3 to 3.1) | Being African American was associated with preferring counselling over medication (OR=2.22, 95% CI: 1.03 to 4.81) | Having a college degree was associated with choosing individual counselling over group counselling (OR=3.0, 95% CI: 1.8 to 5.2) | Being wealthy was associated with choosing active treatment over no treatment (OR=3.74, 95% CI: 1.77 to 7.91) | *NA* |
| Dwight Johnson et al. (2013) | | **Logistic regression models** | *NA* | *NA* | White men were more likely to choose medication compared to Mexican origin men (no further explanation); Mexican men were more likely to accept treatment from a psychiatrist compared to social worker (no further explanation) | *NA* | *NA* | *NA* |
| Groenewoud et al. (2015) | | **Conditional main effects logit** | *NA* | *NA* | *NA* | Respondents with higher education base their choice on a larger number of attributes and they associated a lesser weight to reducing the cost from 80€ to 0€, to increasing the percentage of satisfied patients and to improving the continuity of care | *NA* | *NA* |
| Gum et al. (2006) | | **Multiple logistic regression models** | *NA* | Being female was associated with lower preference for medication (OR=0.71, 95% CI: 0.55 to 0.91) | *No significant findings (OR_minority_=0.86, 95% CI: 0.65 to 1.14)* | *No significant findings (OR_atleasthighschool_=0.94, 95% CI: 0.69 to 1.27)* | *NA* | *NA* |
| Houle et al. (2013) | | **T-test/Chi-square-test and multiple logistic regression** | *No significant findings (t-test, p=0.176)* | Being female was associated with preference for psychotherapy (OR=9.8, 95% CI: 1.9 to 50.0) | *No significant findings (Chi-square-test, p=0.147)* | University level of education was associated with preference for psychotherapy (OR=7.6, 95% CI: 1.6 to 35.4) | *No significant findings (Chi-square-test, p=0.234)* | *NA* |
| Khalsa et al. (2011) | | **Chi-square-test and t-test** | *NA* | *No significant association of gender and preferences* | *No significant association of ethnicity and preferences* | *NA* | *NA* | *NA* |
| Lokkerbol et al. (2019) | | **Random parameters logit and joint models with interaction terms** | *No significant differences between age groups (e.g. preference for face-to-face treatment, p=0.94)* | *NA* | *NA* | Higher educated respondents had stronger aversion against long waiting times (p=0.02) and against fully digital treatment (p=0.02) compared to lower educated respondents | *NA* | *NA* |
| Luck-Sikorski et al. (2017) | | **Logistic regression** | Elderly showed a difficulty in choosing their preferred treatment option | *NA* | *NA* | *NA* | *NA* | *NA* |
| Smith et al. (2021) | | **t-test** | Younger respondents were less likely to indicate that they would use high intensity remote treatment (i.e. videoconferencing) compared with adults (t(68.11)=-2.07, p=0.042, d=0.35)) | *NA* | *NA* | *NA* | *NA* | *NA* |
| *Anxiety* | | | | | | | | |
| Black et al. (2023) | | **t-test** | No significant differences | *NA* | *NA* | *NA* | *NA* | *NA* |
| Lokkerbol et al. (2019) | | **Random parameters logit and joint models with interaction terms** | Younger respondents had less aversion against digital treatment (p=0.01) | *NA* | *NA* | Higher educated respondents had stronger preference for shorter waiting times (p=0.02) and stronger aversion against a treatment intensity of two times per week (p=0.02) | *NA* | *NA* |
|  | | | **Health-related factors** | | | | | |
|  | |  | **Severity of disease** | **Comorbidity** | **Treatment history** | **Family history of disorder** | **Knowledge about counselling** | **Knowledge about medication** |
| *Depression* | | | | | | | | |
| Backenstrass et al. (2006) | | **Chi-square-test** | *No significant differences between major depression and subsyndromal depression (Chi-square-test, p=1.87)* | *NA* | *NA* | *NA* | *NA* | *NA* |
| Dorow et al. (2018) | | **Eight ordinal logistic regression models for all treatment options** | *No significant associations (e.g. preference for psychotherapy, OR_moderate_=1.24, 95% CI: 0.91 to 1.69; reference: mild depression)* | Comorbid GAD was associated with preference for alternative treatment (OR=1.65, 95% CI: 1.17 to 2.34) | Having a treatment history of depression was associated with lower preference for medication (OR=0.51, 95% CI: 0.38 to 0.70) and combined treatment (OR=0.62, 95% CI: 0.45 to 0.84) | *NA* | *NA* |  |
| Dwight Johnson et al. (2000) | | **Three nested logistic regression models (active vs no, med vs counselling, individual vs group)** | *NA* | Having a concurrent 12-month anxiety disorder was associated with preferring active treatment over not treatment (OR=1.74, 95% CI: 1.14 to 2.65) | Having no recent antidepressant treatment was associated with preferring counselling over medication (OR=0.39, 95% CI: 0.27 to 0.56) | *NA* | Greater knowledge about counselling was associated with preferring counselling over medication (OR=2.09, 95% CI: 1.63 to 2.68) and less knowledge of counselling was associated with preferring individual over group counselling (OR=0.61, 95% CI: 0.46 to 0.80) | Greater knowledge about medication was associated with preferring active treatment over no treatment (OR=2.60, 95% CI: 1.55 to 4.36) |
| Dwight Johnson et al. (2010) | | **Logistic regression models** | Symptom severity was associated with treatment choice (no further explanation) | *NA* | Previous counselling was associated with preference for barrier reduction strategies (no further explanation) | *NA* | Knowledge about counselling was associated with treatment choice (no further explanation) | *NA* |
| Gum et al. (2006) | | **Multiple logistic regression models** | Respondents with major depression were more likely to prefer medication than respondents with dysthymia (OR=1.45, 95% CI: 1.12 to 1.86) | *NA* | Respondents who had previously received psychotherapy and found it helpful in the past were less likely to prefer medication (OR=0.59, 95% CI: 0.44 to 0.79) and respondents who had used antidepressants in the past three months (OR=1.74, 95% CI: 1.35 to 2.25) or had found antidepressants helpful in the past (OR=3.10, 95% CI: 1.97 to 4.89) were more likely to prefer medication | *NA* | *NA* | *NA* |
| Houle et al. (2013) | | **Multiple logistic regression** | *NA* | *NA* | *NA* | Family history of depression was associated with preference for psychotherapy (OR=7.8, 95% CI: 1.6 to 37.7) | *NA* | *NA* |
| Khalsa et al. (2011) | | **Chi-squared test and t-test** | *NA* | *NA* | Respondents who preferred psychotherapy reported fewer previous courses of psychotherapy compared to respondents preferring medication (d=0.49, p<0.005) | *NA* | *NA* | *NA* |
| Luck-Sikorski et al. (2017) | | **Logistic regression** | Geriatric Depression Scale (GDS) score > 6 was associated with lower endorsement of psychotherapy (b=-0.26, 95% CI: -0.52 to 0.00), alternative methods (b=-0.34, 95% CI: -0.64 to -0.05), exercise (b=-0.78,95% CI: -1.04 to -0.51) , self-help books (b=-0.30, 95% CI: -0.59 to -0.02) and self-help groups (b=-0.44, 95% CI: -0.74 to -0.15) | *NA* | *NA* | *NA* | *NA* | *NA* |
| *Anxiety* | | | | | | | | |
| Black et al. (2023) | | **t-test** | Respondents who had previously received psychological treatment from mental health professional chose individual face-to-face treatment more often than those who had not (t(94)=22.79, p<0.01, d=0.72) | *NA* | *NA* | *NA* | *NA* | *NA* |

Table S1: Determinants of preferences
