# Supplementary material for: Treatment preferences and their determinants among adults with depression or anxiety in out-patient mental healthcare: systematic review
Source: BJPsych Open. 2025 Oct 1;11(6):e229. doi: 10.1192/bjo.2025.10849 (PMC12529313; doi:10.1192/bjo.2025.10849)
Supplement: Lenz et al. supplementary material 2 — Lenz et al. supplementary material [file S2056472425108491sup002.docx]

**Supplementary Material**

Supplementary Material A: Search Strategy and Search Terms

**Keywords**

preferences, patient preferences, depression, depressive disorder, anxiety, anxiety disorder, treatment.

**PubMed**

| **Number** | **Search string** | **Field** | **Number of results** |
| --- | --- | --- | --- |
| #1 | ("patient preference"[MeSH terms] OR preference* OR patient preference*) | Title/Abstract | 207,958 |
| #2 | ("depressive disorder"[MeSH terms] OR depression OR depressive disorder OR "anxiety disorders"[MeSH terms] OR anxiety) | Title/Abstract | 677,726 |
| #3 | (treatment) | Title/Abstract | 5,485,901 |
| #4 | #1 AND #2 | Title/Abstract | 9,916 |
| #5 | #3 AND #4 | Title/Abstract | **3,990** |

**Web of Science**

| **Number** | **Search string** | **Field** | **Number of results** |
| --- | --- | --- | --- |
| #1 | ("patient preference*" OR preference*) | Topic | 526,935 |
| #2 | (depression OR "depressive disorder*" OR anxiety OR "anxiety disorder*") | Topic | 962,448 |
| #3 | (treatment) | Topic | 6,834,086 |
| #4 | #1 AND #2 | Topic | 16,449 |
| #5 | #3 AND #4 | Topic | **5,998** |

**PsycInfo***mp* ***=*** *title, abstract, heading word, table of contents, key concepts, original title, tests & measures, mesh word*

| **Number** | **Search string** | **Field** | **Number of results** |
| --- | --- | --- | --- |
| #1 | ("patient preference*" OR preference*) | mp | 135,885 |
| #2 | (depression OR "depressive disorder*" OR anxiety OR "anxiety disorder*") | mp | 573,084 |
| #3 | (treatment) (Topic) | mp | 1,363,794 |
| #4 | #1 AND #2 | mp | 8,670 |
| #5 | #3 AND #4 | mp | **2,881** |

**CINAHL**

| **Number** | **Search string** | **Field** | **Number of results** |
| --- | --- | --- | --- |
| #1 | ("patient preference*" OR preference*) | Title or Abstract | 51,693 |
| #2 | (depression OR "depressive disorder*" OR anxiety OR "anxiety disorder*") | Title or Abstract | 215,157 |
| #3 | (treatment) (Topic) | Title or Abstract | 1,058,593 |
| #4 | #1 AND #2 | Title or Abstract | 2,714 |
| #5 | #3 AND #4 | Title or Abstract | **1,166** |

Supplementary Material B: Quality Assessment

Study quality was evaluated with the Mixed Methods Appraisal Tool (Hong, Q.N., Fàbregues, S., Bartlett, G., Boardman, F., Cargo, M., Dagenais, P., Gagnon, M.P., Griffiths, F., Nicolau, B., O'Cathain, A., Rousseau, M.C., Vedel, I., Pluye, P., 2018. The Mixed Methods Appraisal Tool (MMAT) version 2018 for information professionals and researchers. Educ Inform 34, 285-291.)

| Quantitative descriptive studies | | | | | |
| --- | --- | --- | --- | --- | --- |
|  | **4.1.** | **4.2.** | **4.3.** | **4.4.** | **4.5.** |
|  | **Is the sampling strategy relevant to adress the research question?** | **Is the sample representative of the target population?** | **Are the measurements appropriate?** | **Is the risk of nonresponse bias low?** | **Is the statistical analysis appropriate to answer the research question?** |
| Backenstrass 2006 | Yes | Yes | No | No | No |
| Basile 2024 | No | No | Yes | No | No |
| Black 2023 | No | No | Yes | No | No |
| Boehlen 2016 | Yes | Yes | No | No | No |
| Dorow 2018 | Yes | Yes | Yes | No | Yes |
| Dwight Johnson 2000 | Yes | Yes | No | Yes | Yes |
| Dwight Johnson 2010 | Yes | No | Yes | No | Yes |
| Dwight Johnson 2013 | Yes | Yes | Yes | No | Yes |
| Groenewoud 2015 | Yes | Yes | Yes | Yes | Yes |
| Gum 2006 | Yes | Yes | No | Yes | Yes |
| Houle 2013 | No | Yes | No | No | No |
| Khalsa 2011 | Yes | Yes | No | No | No |
| Lokkerbol 2019 Depression | No | No | Yes | No | Yes |
| Lokkerbol 2019 Anxiety | No | No | Yes | No | Yes |
| Luck-Sikorski 2017 | Yes | Yes | Yes | No | Yes |
| Muntingh 2019 | Yes | Yes | Yes | No | Yes |
| Raue 2009 | Yes | Yes | No | Yes | No |
| Smith 2021 | No | No | Yes | No | No |
| Tompkins 2017 | Yes | Yes | Yes | No | No |
|  |  |  |  |  |  |
| Mixed methods studies | |  |  |  |  |
|  | **5.1.** | **5.2.** | **5.3.** | **5.4.** | **5.5.** |
|  | **Is there an adequate rationale for using a mixed methods design to address the research question?** | **Are the different components of the study effectively integrated to answer the research question?** | **Are the outputs of the integration of qualitative and quantitative components adequately interpreted?** | **Are divergences and inconsistencies between quantitative and qualitative results adequately addressed?** | **Do the different components of the study adhere to the quality criteria of each tradition of the methods involved?** |
| Löwe 2006 | Yes | Yes | Yes | Can't tell | Yes |
| Soucy 2017 | No | Yes | Yes | Yes | No |

Supplementary Material C: Overview of determinants

The table lists all determinants (significant and not significant) that were examined in each study. Those that were significant are printed in bold. Selected determinants that were significant in at least one study are further explained in Table 3 in the article.

| **Author (Year)** | **Investigated determinants** | | |
| --- | --- | --- | --- |
|  | **Sociodemographic** | **Health-related** | **Other** |
| Backenstrass et al. (2006) | - | **Severity of disease** | - |
| Black et al. (2023) | **Age**, geographical location | **Treatment history** | - |
| Dorow et al. (2018) | **Age**, **gender**, **marital status**, **educational level**, vocational qualification | Severity of depression, **treatment history**, **comorbid panic or anxiety disorder**, HRQOL | Empowerment |
| Dwight Johnson et al. (2000) | **Gender**, **education**, **age**, **ethnicity**, **wealth**, number of young children in household | Ever taken medication for emotional problems, ever had counselling, current depressive disorder and recent treatment, symptoms only and recent treatment, **antidepressant in the last 6 months**, counselling in the last 6 months, **medication knowledge**, **counselling knowledge**, CES-D score, disorder status, **anxiety disorder**, number of chronic diseases, SF-36 MCS, SF-36 PCS | Paid sick leave, stigma associated with psychiatric care |
| Dwight Johnson et al. (2010) | Gender, employment status, age, years of education, language spoken | Referral source, **baseline depressive symptom severity**, probable anxiety disorder, number of comorbid medical illnesses, **past treatment**, **knowledge about medication and counselling** | Insurance status, stigma |
| Dwight Johnson et al. (2013) | **Ethnicity** | - | - |
| Groenewoud et al. (2015) | **Education** | Severity of disease, choice profile (outcome-focused vs trust-focused) | - |
| Gum et al. (2006) | **Gender**, ethnic minority, **education** | **Severity of depression**, SCL-20 depression score, cognitive impairment, prior antidepressant medication experience, **antidepressant use in the past 3 months**, **prior counselling experience**, any specialty mental health visit or psychotherapy in the past 3 months | - |
| Houle et al. (2013) | Age, **gender**, **education**, employment status, income, ethnicity | **Family member with depression**, psychiatric comorbidity, current preferred treatment, current antidepressant treatment, current psychotherapy, depression severity, illness representations, depression attributions, treatment acceptability | - |
| Khalsa et al. (2011) | Gender, ethnicity | **Previous experience with psychotherapy**, previous experience with medication | - |
| Lokkerbol et al. (2019a) Depression | Age, **education** | Level of impairment |  |
| Lokkerbol et al. (2019b) Anxiety | **Age**, **education** | Level of impairment | - |
| Luck-Sikorski et al. (2017) | Age | **Severity of depression** | - |
| Smith et al. (2021) | **Age**, geographical location | - | - |

Supplementary Material D: Completed PRISMA Checklist

| **Section and Topic** | **Item #** | **Checklist item** | **Location where item is reported** |
| --- | --- | --- | --- |
| **TITLE** | | |  |
| Title | 1 | Identify the report as a systematic review. | p. 1 |
| **ABSTRACT** | | |  |
| Abstract | 2 | See the PRISMA 2020 for Abstracts checklist. | p. 2 |
| **INTRODUCTION** | | |  |
| Rationale | 3 | Describe the rationale for the review in the context of existing knowledge. | p. 3 |
| Objectives | 4 | Provide an explicit statement of the objective(s) or question(s) the review addresses. | p. 3 |
| **METHODS** | | |  |
| Eligibility criteria | 5 | Specify the inclusion and exclusion criteria for the review and how studies were grouped for the syntheses. | p. 4 |
| Information sources | 6 | Specify all databases, registers, websites, organisations, reference lists and other sources searched or consulted to identify studies. Specify the date when each source was last searched or consulted. | p. 4 |
| Search strategy | 7 | Present the full search strategies for all databases, registers and websites, including any filters and limits used. | Supp. Mat. A |
| Selection process | 8 | Specify the methods used to decide whether a study met the inclusion criteria of the review, including how many reviewers screened each record and each report retrieved, whether they worked independently, and if applicable, details of automation tools used in the process. | p. 4 |
| Data collection process | 9 | Specify the methods used to collect data from reports, including how many reviewers collected data from each report, whether they worked independently, any processes for obtaining or confirming data from study investigators, and if applicable, details of automation tools used in the process. | p. 4 |
| Data items | 10a | List and define all outcomes for which data were sought. Specify whether all results that were compatible with each outcome domain in each study were sought (e.g. for all measures, time points, analyses), and if not, the methods used to decide which results to collect. | p. 4 |
|  | 10b | List and define all other variables for which data were sought (e.g. participant and intervention characteristics, funding sources). Describe any assumptions made about any missing or unclear information. | p. 4 |
| Study risk of bias assessment | 11 | Specify the methods used to assess risk of bias in the included studies, including details of the tool(s) used, how many reviewers assessed each study and whether they worked independently, and if applicable, details of automation tools used in the process. | p. 4 |
| Effect measures | 12 | Specify for each outcome the effect measure(s) (e.g. risk ratio, mean difference) used in the synthesis or presentation of results. | p. 4 |
| Synthesis methods | 13a | Describe the processes used to decide which studies were eligible for each synthesis (e.g. tabulating the study intervention characteristics and comparing against the planned groups for each synthesis (item #5)). | p. 4 |
|  | 13b | Describe any methods required to prepare the data for presentation or synthesis, such as handling of missing summary statistics, or data conversions. | p. 4 |
|  | 13c | Describe any methods used to tabulate or visually display results of individual studies and syntheses. | p. 4 |
|  | 13d | Describe any methods used to synthesize results and provide a rationale for the choice(s). If meta-analysis was performed, describe the model(s), method(s) to identify the presence and extent of statistical heterogeneity, and software package(s) used. | p.4 |
|  | 13e | Describe any methods used to explore possible causes of heterogeneity among study results (e.g. subgroup analysis, meta-regression). | p. 4 |
|  | 13f | Describe any sensitivity analyses conducted to assess robustness of the synthesized results. | NA |
| Reporting bias assessment | 14 | Describe any methods used to assess risk of bias due to missing results in a synthesis (arising from reporting biases). | NA |
| Certainty assessment | 15 | Describe any methods used to assess certainty (or confidence) in the body of evidence for an outcome. | NA |
| **RESULTS** | | |  |
| Study selection | 16a | Describe the results of the search and selection process, from the number of records identified in the search to the number of studies included in the review, ideally using a flow diagram. | p. 5 |
|  | 16b | Cite studies that might appear to meet the inclusion criteria, but which were excluded, and explain why they were excluded. | p. 5 |
| Study characteristics | 17 | Cite each included study and present its characteristics. | p. 6, Table 1 |
| Risk of bias in studies | 18 | Present assessments of risk of bias for each included study. | p. 6, Table 1, Sup. Mat B |
| Results of individual studies | 19 | For all outcomes, present, for each study: (a) summary statistics for each group (where appropriate) and (b) an effect estimate and its precision (e.g. confidence/credible interval), ideally using structured tables or plots. | Tables 2-3 |
| Results of syntheses | 20a | For each synthesis, briefly summarise the characteristics and risk of bias among contributing studies. | p. 8-11, p. 15-16 |
|  | 20b | Present results of all statistical syntheses conducted. If meta-analysis was done, present for each the summary estimate and its precision (e.g. confidence/credible interval) and measures of statistical heterogeneity. If comparing groups, describe the direction of the effect. | Tables 2-3 |
|  | 20c | Present results of all investigations of possible causes of heterogeneity among study results. | Tables 1-3 |
|  | 20d | Present results of all sensitivity analyses conducted to assess the robustness of the synthesized results. | NA |
| Reporting biases | 21 | Present assessments of risk of bias due to missing results (arising from reporting biases) for each synthesis assessed. | NA |
| Certainty of evidence | 22 | Present assessments of certainty (or confidence) in the body of evidence for each outcome assessed. | NA |
| **DISCUSSION** | | |  |
| Discussion | 23a | Provide a general interpretation of the results in the context of other evidence. | p. 24 |
|  | 23b | Discuss any limitations of the evidence included in the review. | p. 24-25 |
|  | 23c | Discuss any limitations of the review processes used. | p. 25 |
|  | 23d | Discuss implications of the results for practice, policy, and future research. | p. 24-25 |
| **OTHER INFORMATION** | | |  |
| Registration and protocol | 24a | Provide registration information for the review, including register name and registration number, or state that the review was not registered. | p. 4 |
|  | 24b | Indicate where the review protocol can be accessed, or state that a protocol was not prepared. | p. 4 |
|  | 24c | Describe and explain any amendments to information provided at registration or in the protocol. | p. 4 |
| Support | 25 | Describe sources of financial or non-financial support for the review, and the role of the funders or sponsors in the review. | p. 26 |
| Competing interests | 26 | Declare any competing interests of review authors. | p. 26 |
| Availability of data, code and other materials | 27 | Report which of the following are publicly available and where they can be found: template data collection forms; data extracted from included studies; data used for all analyses; analytic code; any other materials used in the review. | p. 26 |

*From:*  Page MJ, McKenzie JE, Bossuyt PM, Boutron I, Hoffmann TC, Mulrow CD, et al. The PRISMA 2020 statement: an updated guideline for reporting systematic reviews. BMJ 2021;372:n71. doi: 10.1136/bmj.n71. This work is licensed under CC BY 4.0. To view a copy of this license, visit <https://creativecommons.org/licenses/by/4.0/>
